# LncRNAs in Ovarian Cancer: Emerging Insights and Future Perspectives in Tumor Biology and Clinical Applications

**DOI:** 10.3390/cancers18030484

**Published:** 2026-02-01

**Authors:** Michaela A. Boti, Marios A. Diamantopoulos, Sevastiana Charalampidou, Andreas Scorilas

**Affiliations:** Department of Biochemistry and Molecular Biology, Faculty of Biology, National and Kapodistrian University of Athens, Panepistimiopolis, 15701 Athens, Greece; miboti@biol.uoa.gr (M.A.B.); imdiamantop@biol.uoa.gr (M.A.D.); sevichara@biol.uoa.gr (S.C.)

**Keywords:** ovarian cancer, long non-coding RNAs (lncRNAs), gene expression regulation, cancer pathogenesis, biomarkers, therapeutic targets, long-read sequencing, third-generation sequencing, therapeutics, CRISPR-Cas13

## Abstract

**Background/Objectives**: Ovarian cancer (OC) remains one of the most lethal gynecological malignancies, mainly because it is frequently diagnosed at advanced stages due to nonspecific symptoms and the lack of effective screening strategies. Long non-coding RNAs (lncRNAs) have emerged as key regulators of gene expression, and accumulating evidence implicates them in OC initiation, progression, and treatment response. This review aims to comprehensively summarize the molecular mechanisms of lncRNAs in OC, examine their clinical potential as biomarkers, and discuss emerging technologies that are about to advance lncRNA research and therapeutics in OC. **Methods**: A comprehensive review of published studies investigating lncRNA expression, function, and clinical relevance in OC was conducted. Mechanistic insights were integrated across multiple regulatory levels, including epigenetic, transcriptional, post-transcriptional, and post-translational control. Advances in transcriptomic technologies and RNA-targeting techniques were also examined. **Results**: LncRNAs influence OC through diverse mechanisms, including chromatin remodeling, transcriptional regulation, RNA splicing, mRNA stability, protein modulation, competing endogenous RNA networks, and nuclear organization. Their dysregulation is linked to tumor progression, metastasis, chemoresistance, and poor patient outcomes. Numerous lncRNAs exhibit diagnostic and prognostic value, underscoring their clinical potential. Advances in long-read sequencing have improved lncRNA annotation and isoform resolution, while CRISPR-Cas13 offers a potential approach for selective RNA-targeted therapy. **Conclusions**: LncRNAs are critical molecules in OC development and progression, holding potential in advancing OC diagnosis, prognosis, and treatment. Continued integration of functional studies, advanced sequencing technologies, and RNA-targeting approaches can facilitate the clinical translation of lncRNAs for early OC diagnosis and management.

## Simple Summary

Ovarian cancer is a highly lethal disease, often diagnosed at advanced stages, a fact that complicates treatment. Long non-coding RNAs (lncRNAs) are RNA molecules that do not encode proteins but play crucial roles in gene expression regulation. This review focuses on the roles of lncRNAs, giving mechanistic insights from ovarian cancer-related studies, and highlights their potential utility as diagnostic, prognostic, and predictive indicators of the disease. By discussing emerging insights from cutting-edge technologies, including long-read sequencing and CRISPR-Cas13 system, this review aims to provide a framework for future lncRNA research and therapeutics in ovarian cancer. The findings could provide an in-depth understanding of lncRNAs’ dysregulation in this malignancy and potentially accelerate the future translation of lncRNA-based strategies into clinical practice for ovarian cancer diagnosis and treatment.

## 1. Introduction

Ovarian cancer (OC) is the eighth most commonly diagnosed malignancy among women worldwide, with 324,603 new cases reported in 2022 according to the Global Cancer Observatory [1]. Despite its lower incidence compared to breast cancer, OC is characterized by a higher mortality rate and remains one of the leading causes of gynecological cancer-related death. Epidemiological projections suggest that, without significant advances in early detection and therapeutic strategies, global mortality from OC is expected to increase by nearly 70% by 2050. This poor prognosis is primarily attributable to the disease’s insidious clinical course [2]. Early-stage OC is frequently asymptomatic or presents with nonspecific symptoms, and the lack of effective population-based screening approaches results in delayed diagnosis [3,4]. As a consequence, most patients are diagnosed at advanced stages, when treatment options are limited and long-term survival rates are low, leading to OC being widely referred to as a “silent killer” [5,6].

Long non-coding RNAs (lncRNAs) have emerged as key regulators of gene expression and are increasingly recognized for their involvement in cancer pathogenesis. LncRNAs are conventionally defined as transcripts longer than 200 nucleotides that lack protein-coding potential [7], although more stringent classifications describe them as RNA polymerase II-transcribed transcripts typically exceeding 500 nucleotides. This refined definition facilitates their distinction from shorter non-coding RNA species, like small nuclear RNAs and intron-derived small nucleolar RNAs [8]. Accumulating evidence demonstrates that lncRNAs regulate gene expression at multiple levels [9]. Through interactions with chromatin-modifying complexes, transcription factors, RNA polymerase, and other RNA molecules, lncRNAs influence epigenetic regulation, transcriptional control, and post-transcriptional processing [10,11,12]. By coordinating these regulatory networks, lncRNAs play critical roles in maintaining normal function [13], and their dysregulation contributes to tumor initiation, progression, metastasis, and therapeutic resistance across multiple cancer types [14,15,16,17,18].

In OC, numerous lncRNAs are aberrantly expressed relative to normal ovarian epithelium and have been implicated in key oncogenic processes, including epithelial–mesenchymal transition (EMT), DNA damage response, angiogenesis, and proliferation, acting as either oncogenes or tumor suppressors [18,19,20]. These findings highlight lncRNAs as integral components of OC biology and support their investigation as potential diagnostic, prognostic, and predictive biomarkers, as well as therapeutic targets [21,22].

Recent advances in transcriptomics and RNA-targeting technologies are rapidly transforming lncRNA research in cancer, including OC, allowing more accurate transcript characterization and paving the way for the development of novel therapeutic strategies. Third-generation sequencing platforms (TGS) have enabled the sequencing of full-length transcripts, overcoming the limitations of short-read sequencing in resolving lncRNA isoform diversity and structural complexity [23,24,25,26]. In parallel, emerging RNA-targeting technologies like CRISPR-Cas13 system have expanded the toolkit for lncRNA functional studies by enabling selective and reversible RNA degradation without altering genomic DNA [27]. Beyond its utility as a versatile tool for modulating gene expression in research, CRISPR-Cas13 holds substantial therapeutic potential in OC, offering a strategy for the targeted silencing of oncogenic lncRNAs and supporting future clinical translation [28].

In this review, we summarize the current understanding of lncRNAs in OC, focusing on their mechanisms of action, roles in tumor initiation and progression, and impact on malignant phenotypes. We also discuss emerging evidence supporting the clinical potential of lncRNAs as diagnostic, prognostic, and predictive biomarkers, as well as therapeutic targets in OC. Finally, we highlight recent technological advances that are reshaping the field—including long-read sequencing and RNA-targeting CRISPR-Cas13 system—and outline future directions in lncRNA research and therapeutics in OC.

## 2. Molecular Mechanisms and Functional Insights of LncRNAs in OC

**Figure 1:**
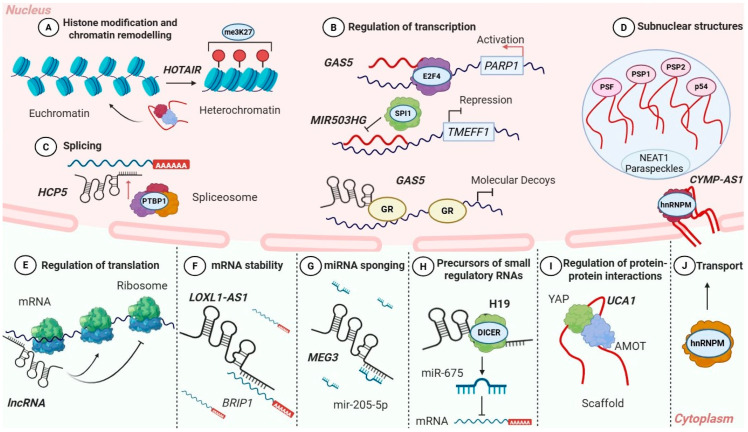
Molecular mechanisms of lncRNA-mediated regulation in OC. Schematic overview of the diverse molecular mechanisms through which lncRNAs regulate gene expression in OC. In the nucleus, lncRNAs (**A**) modulate chromatin organization and histone modifications (e.g., *HOTAIR*), (**B**) interact with transcription factors (GAS5, *MIR503HG*), (**C**) influence alternative splicing (*HCP5*), and (**D**) contribute to the formation of subnuclear structures such as paraspeckles (*NEAT1*). In the cytoplasm, lncRNAs control mRNA (**E**) translation and (**F**) stability (*LOXL1-AS1*), (**G**) act as competing endogenous RNAs by sponging miRNAs (*MEG3*), (**H**) serve as precursors for small regulatory RNAs (*H19*), (**I**) function as molecular scaffolds (*UCA1*) regulating protein interactions, and (**J**) participate in intracellular transport processes (*CYMP-AS1*).

LncRNAs have emerged as critical regulators of gene expression, acting through a wide spectrum of molecular mechanisms across multiple levels of gene regulation (Figure 1) [29,30]. Under physiological conditions, lncRNAs exert their functions by interacting with chromatin-modifying complexes, transcriptional machinery, RNA-binding proteins, and signaling components, thus modulating chromatin organization, transcription, RNA processing, protein stability, and intracellular signaling [30,31,32]. Dysregulation of these mechanismsprofoundly alters gene regulatory networks and contributes to malignant transformation [33,34]. In OC, aberrant expression or activity of lncRNAs reprograms these fundamental regulatory processes, promoting tumor initiation, progression, EMT, metastasis, drug resistance, and adaptation to cellular stress [35,36]. This section describes the principal molecular mechanisms of lncRNA action and integrates mechanistic insights derived from OC studies. Emphasis is placed on epigenetic regulation, transcriptional and post-transcriptional control, protein regulation, ceRNA networks, nuclear architecture, and the generation of small regulatory RNAs.

### 2.1. Epigenetic Regulation: Histone/DNA Modification and Chromatin Remodeling

One of the most extensively studied functions of lncRNAs is their role in epigenetic regulation [37]. Evidence from studies in various cancer types demonstrates that lncRNAs play critical roles in controlling gene expression by modulating chromatin structure and regulating the recruitment and activity of chromatin-modifying complexes (Figure 1) [38]. Through these interactions, lncRNAs contribute to the activation or repression of specific chromatin regions.

In general, lncRNAs can function either in *cis*, regulating genes located in close genomic proximity, or in *trans*, affecting gene expression at distant loci across the genome [39,40,41]. In many cases, lncRNAs act as molecular guides or scaffolds that facilitate the targeting of epigenetic regulators, like Polycomb repressive complex 2 (PRC2), histone acetyltransferases, histone deacetylases, or DNA methyltransferases to specific genomic regions [42,43]. Rather than possessing intrinsic enzymatic activity, lncRNAs exert their regulatory effects by coordinating these protein complexes and directing them to defined chromatin sites [44]. Through this mechanism, lncRNAs indirectly influence histone modifications and DNA methylation patterns, thus promoting transcriptional activation or repression. A notable example is XIST, which is involved in X-chromosome inactivation and imprinting, illustrating lncRNAs’ capacity to achieve large-scale chromatin reorganization [42,45]. Although many of these epigenetic mechanisms have been extensively characterized in multiple cancer types, the specific lncRNA–chromatin interactions described in Section LncRNAs as Regulators of Epigenetic Marks in OC are supported by experimental evidence in OC.

#### LncRNAs as Regulators of Epigenetic Marks in OC

The lncRNA HOTAIR is one of the best-characterized examples in OC. Functioning as a molecular scaffold, it recruits PRC2 and LSD1 chromatin-modifying complexes to specific genomic loci, leading to H3K27 trimethylation or H3K4 demethylation and transcriptional silencing of tumor suppressor genes. Mechanistically, HOTAIR interacts with the enhancer of zeste homolog 2 (EZH2), a core component of PRC2, facilitating its targeting to chromatin and H3K27 trimethylation (Figure 1A) [46,47]. On the other hand, HOTAIR associates with the LSD1 complex, enabling coordinated histone demethylation. Notably, the PRC2- and LSD1-interacting domains contribute both cooperatively and independently to HOTAIR’s regulatory activity. While truncation of the LSD1-binding domain preserves PRC2-dependent transcriptional reprogramming, it is insufficient to sustain HOTAIR-mediated promotion of cell migration [48].

A key mechanistic theme emerging from these studies is the participation of multiple oncogenic lncRNAs on PRC2/EZH2-mediated epigenetic repression. For example, LINC00511 also promotes OC cell proliferation by interacting with EZH2 [49]. This lncRNA directly interacts with EZH2 and facilitates epigenetic repression of the cyclin-dependent kinase inhibitor *p21*, through H3K27 trimethylation. Correspondingly, depletion of LINC00511 or EZH2 leads to upregulation of p21 expression and attenuation of OC cell proliferation [18]. These findings indicate that LINC00511 acts as a nuclear scaffold guiding PRC2-mediated epigenetic silencing of p21, thereby promoting cell cycle progression in OC cells.

Similarly, other oncogenic lncRNAs like TP73-AS1, LINC01210, and LINC00702, have been shown to be involved in the same pathway by recruiting PRC2 to promoters of differentiation- and growth-associated genes, sustaining oncogenic transcriptional programs. TP73-AS1 promotes proliferation and invasion while suppressing apoptosis in OC. It epigenetically represses p21 by recruiting EZH2 to its promoter, increasing H3K27 trimethylation, as confirmed by chromatin immunoprecipitation and luciferase reporter assays [50]. LINC01210 negatively regulates KLF4 by interacting with EZH2 and facilitating its recruitment to the *KLF4* promoter, resulting in H3K27 trimethylation and transcriptional repression. RNA immunoprecipitation and chromatin immunoprecipitation assays confirm the direct interaction and promoter targeting, and depletion of KLF4 reverses the effects of LINC01210 knockdown on cell proliferation, migration, and invasion [51]. LINC00702 is similarly interacting with EZH2 to suppress *KLF2* transcription via H3K27 trimethylation, promoting OC cell proliferation [52].

Together, these examples demonstrate that PRC2/EZH2-mediated chromatin repression is a central epigenetic mechanism exploited by multiple lncRNAs in OC (Table 1), reinforcing the importance of this pathway in tumor progression and highlighting PRC2/EZH2 as a common therapeutic target for future interventions.

### 2.2. Transcriptional Regulation: Interactions with Transcriptional Machinery

Beyond chromatin remodeling, lncRNAs can directly regulate transcription by interacting with components of the transcriptional machinery (Figure 1) [53]. They influence gene expression by modulating the recruitment, activity, or stability of RNA polymerase II and transcription factors at promoters and enhancers [54]. Enhancer-associated lncRNAs, often referred to as enhancer RNAs (eRNAs), are transcribed from active enhancer regions and facilitate enhancer–promoter looping, thus promoting transcription of target genes [55,56]. Although the enhancer-mediated function of eRNAs is well established in other malignancies, their specific relevance in OC requires further experimental validation. Numerous eRNAs have been identified in OC cell lines and patient specimens, but their functional roles in this malignancy remain unknown [57,58].

LncRNAs can repress transcription by preventing the binding of RNA polymerase II or transcription factors to regulatory regions, either through transcriptional interference or by altering the local availability of these factors [53,54]. These lncRNAs act as decoys, preventing transcription factors to interact with DNA and suppressing target gene’s expression (Figure 1). This mechanism has been studied in multiple cancer types, including OC, underscoring its significance in gene regulation.

#### LncRNAs as Regulators of Transcription in OC

In OC, multiple lncRNAs have been reported to function as direct regulators of transcription, either by activating oncogenes or by promoting the repression of tumor suppressor genes. The tumor-suppressive lncRNA GAS5 recruits the transcription factor E2F4 to the *PARP1* promoter, enhancing its transcription and increasing chemosensitivity in OC cell lines (Figure 1B) [59].

Similarly, MIR503HG, which is markedly downregulated in OC tissues, exerts tumor-suppressive effects through transcriptional repression. MIR503HG is predominantly localized in the nucleus where it binds to the transcription factor SPI1, preventing its association with the *TMEFF1* promoter (Figure 1B). This interaction results in the suppression of *TMEFF1* transcription. Loss of MIR503HG leads to increased *TMEFF1* expression, activation of the MAPK and PI3K/AKT signaling pathways, and enhanced proliferation, invasion, and EMT. Importantly, genetic or pharmacological inhibition of *TMEFF1* reverses these effects, confirming its role as a key downstream effector of MIR503HG [60].

The tumor-suppressive lncRNA FAM225B and its downstream target PDIA4 are downregulated in OC cell lines and in the serum of patients with OC. RNA pull-down, chromatin immunoprecipitation (ChIP), and RNA immunoprecipitation (RIP) assays demonstrate that FAM225B directly interacts with the RNA-binding protein DDX17 and facilitates its recruitment to the *PDIA4* promoter, thereby enhancing *PDIA4* transcription [61]. Loss of *PDIA4* abolishes the tumor-suppressive effects of FAM225B, highlighting the importance of the FAM225B/DDX17/PDIA4 axis in OC progression.

**Figure 2:**
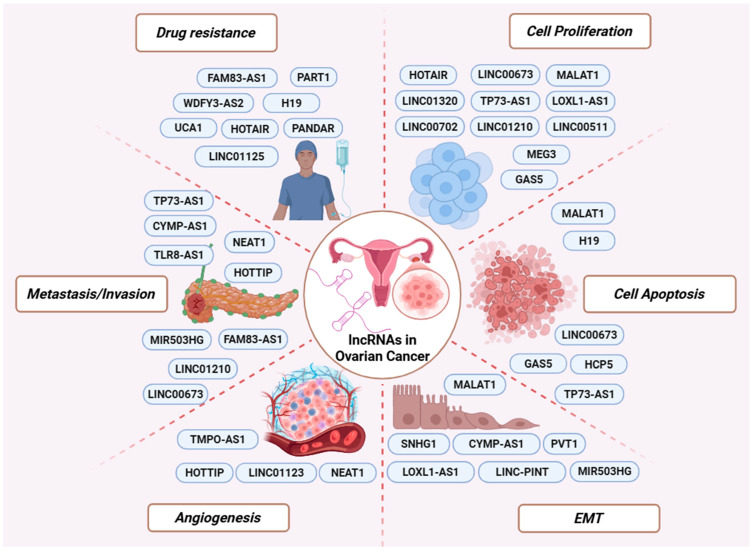
LncRNAs involved in major cancer-related processes. Representative oncogenic and tumor-suppressive lncRNAs are depicted according to their involvement in key aspects of OC biology, including drug resistance, cell proliferation, apoptosis, EMT, angiogenesis, and metastasis/invasion.

In contrast, several oncogenic lncRNAs promote OC progression by repressing the transcription of tumor suppressor genes. LINC01320 is overexpressed in OC tissues and promotes cell proliferation and metastasis (Figure 2). It functions by recruiting the transcriptional repressor purine-rich element binding protein B (PURB) to the *DDB2* promoter, leading to transcriptional silencing of *DDB2*. This repression results in increased NEDD4L expression, disruption of TGF-β/SMAD signaling, and accelerated tumor progression [62].

LncRNA TMPO-AS1 also contributes to tumor progression through transcriptional regulation. TMPO-AS1 interacts with the transcriptional repressor E2F6 and prevents its binding to the *LCN2* promoter, thus enhancing *LCN2* transcription. Upregulation of LCN2 leads to enhanced tumorigenesis and angiogenesis in OC cells. Restoration of E2F6 or *LCN2* overexpression reverses the aggressive phenotypes induced by TMPO-AS1, supporting a TMPO-AS1/E2F6/LCN2 regulatory axis in OC [63].

Additional transcriptional regulatory mechanisms have been described. The tumor-suppressive lncRNA RFPL1S-202 enhances chemosensitivity and suppresses metastasis by interacting with DEAD-Box Helicase 3 X-linked (DDX3X), increasing m6A modification of *IFNB1* mRNA and consequently reducing STAT1 phosphorylation. This reduces STAT1 phosphorylation and downstream interferon-related gene expression [64]. Finally, LINC00176 functions as a molecular scaffold that facilitates the interaction between BCL3 and the NF-κB p50 subunit at the *CP* promoter, activating ceruloplasmin transcription and promoting oncogenic NF-κB signaling in OC cells [65].

Overall, lncRNAs that regulate transcription in OC modulate key oncogenic pathways, including PI3K/AKT, MAPK, NF-κB, and TGF-β/SMAD. Within this framework, distinct lncRNAs exert opposing functional effects: some promote tumor progression by activating pro-survival signaling, whereas others act as tumor-suppressors by inhibiting these pathways and enhancing apoptotic programs. Together, these examples highlight the central role of lncRNA networks in controlling key signaling pathways related to OC biology and, thus, drive or restrain cancer pathogenesis and progression.

### 2.3. Post-Transcriptional Regulation: RNA Processing and Alternative Splicing

LncRNAs play critical roles in post-transcriptional regulation, particularly in RNA processing and alternative splicing (Figure 1) [32]. Based on studies in diverse cellular systems and multiple cancer types, many lncRNAs are known to localize in the nucleus, where they interact with splicing factors and RNA-binding proteins to modulate splice-site selection and exon inclusion or exclusion, and transcript maturation [11,32,66].

During splicing, lncRNAs regulate RNA processing through diverse mechanisms [67]. Some form RNA-DNA hybrids at their genomic loci, inducing transcriptional pausing or altering splicing factor recruitment to nascent transcripts. Others engage in RNA-RNA interactions with pre-mRNAs, masking or exposing splice sites and regulatory elements, thus directly controlling exon inclusion or exclusion [67]. Another important mechanism involves the regulation of splicing factors’ activity, localization, and availability [9,68]. For this function, lncRNAs act as molecular scaffolds that assemble splicing regulators into ribonucleoprotein complexes or as decoys that sequester splicing factors away from their target transcripts. Moreover, they can promote their activation or repression by modifications [67,68]. Although these regulatory principles are broadly conserved, their specific functional relevance in OC has been elucidated only for a subset of lncRNAs.

#### Splicing-Related LncRNAs in OC

In OC, a growing body of evidence supports a direct role for specific lncRNAs in alternative splicing regulation, with functional consequences in cell survival, metastasis, and chemoresistance. MALAT1 is among the most extensively studied splicing-associated lncRNAs. Its elevated expression in anoikis-resistant OC cells promotes the alternative splicing of *KIF1B*, a pro-apoptotic gene, through modulation of the splicing factor RBFOX2. Knockdown of MALAT1 reduces RBFOX2 and favors the pro-apoptotic KIF1B-β isoform, increasing anoikis sensitivity and susceptibility to cell death [69].

In addition to MALAT1, another oncogenic lncRNA that acts as a splicing regulator is the recently identified HCP5. HCP5 is upregulated in OC tissues and cell lines, and its silencing significantly reduces cell viability, induces apoptosis, and promotes G0/G1 cell cycle arrest. RNA pull-down and RNA RIP assays demonstrate that HCP5 interacts with the splicing factor PTBP1 (Figure 1C), enhancing its activity and promoting cancer cell survival. Notably, overexpression of HCP5 partially rescues the effects of PTBP1 knockdown, highlighting the functional relevance of this interaction [70].

The p53-associated lncRNA PANDAR has been identified as a key molecule in cisplatin resistance. PANDAR is highly expressed in cisplatin-resistant OC tissues and cells, but not in cisplatin-sensitive ones, and this expression is dependent on wild-type p53. Overexpression of PANDAR promotes cell survival and tumor growth during cisplatin treatment, while its silencing reduces tumor growth. Mechanistically, PANDAR interacts with the splicing factor SFRS2, which negatively regulates p53 activity by inhibiting its phosphorylation at serine 15. This interaction reduces p53-mediated activation of pro-apoptotic genes like *PUMA*, forming a PANDAR-SFRS2-p53 feedback loop that promotes chemoresistance [71].

Collectively, these studies demonstrate that although many splicing-related lncRNA mechanisms have been initially described in other cancer types, a subset is now well validated in OC. LncRNAs that regulate alternative splicing frequently act through a limited set of splicing factors, including RBFOX2, PTBP1, and SFRS2, thereby influencing apoptotic signaling, anoikis resistance, and chemotherapy response (Table 1).

### 2.4. Post-Transcriptional Control of mRNA Stability and Translation

In the cytoplasm, lncRNAs exert extensive control over post-transcriptional gene regulation by influencing mRNA stability [72], subcellular localization [30], and translational efficiency (Figure 1) [73]. Through direct interactions with mRNAs or by associating with RNA-binding proteins, lncRNAs can protect transcripts from degradation or promote their decay, thereby regulating protein levels independently of transcription [72,74].

LncRNAs also influence translation by controlling mRNA localization [30,73]. By directing transcripts to specific subcellular compartments with differing capacity to support protein synthesis, lncRNAs can promote or repress translation [75]. Dynamic changes in lncRNA localization in response to cellular signals further enable dynamic control of protein synthesis.

Beyond spatial regulation, lncRNAs also modulate translation through direct interactions with the translational machinery [76]. In other cancer types, lncRNAs have been shown to bind translation factors or ribosomal components, thereby enhancing or inhibiting ribosome recruitment to target mRNAs (Figure 1E) [30]. Although this mechanism has not yet been specifically characterized in OC, it underscores the capability of lncRNAs to interact with diverse components of the translational machinery, enabling rapid and reversible control of protein synthesis.

#### LncRNAs Modulating mRNA Stability and Protein Synthesis in OC

A prime example of post-transcriptional regulation in OC is LOXL1-AS1. LOXL1-AS1 is significantly overexpressed in OC tissues and cell lines and promotes OC progression. Silencing LOXL1-AS1 reduces cell proliferation, invasion, and EMT in vitro and in vivo. Mechanistically, LOXL1-AS1 stabilizes *BRIP1* mRNA (Figure 1F), and overexpression of BRIP1 reverses the inhibitory effects of LOXL1-AS1 knockdown, indicating that LOXL1-AS1 promotes tumorigenesis by enhancing mRNA stability [77].

Similarly, TLR8-AS1 is upregulated in OC and plays a role in metastasis and chemoresistance. More precisely, TLR8-AS1 stabilizes *TLR8* mRNA, leading to increased expression of TLR8 and the activation of NF-κB signaling, which in turn enhances OC cell invasion and resistance to chemotherapy (Figure 2) [78].

Another key lncRNA, HOTTIP, is highly expressed in OC cells, particularly under hypoxic conditions. A recent study has shown that HOTTIP promotes cell migration, invasion, and viability by forming a positive feedback loop with HIF-1α, stabilizing its expression. This interaction increases VEGF secretion, promotes angiogenesis, and supports aggressive tumor behavior. Notably, knockdown of HOTTIP reduces HIF-1α levels and cell metastasis while enhancing apoptosis, suggesting that HOTTIP plays a key role in OC progression under hypoxic conditions [79].

The described findings demonstrate that cytoplasmic lncRNAs in OC primarily promote tumor progression through mRNA stabilization, enhancing oncogenic signaling pathways, including NF-κB and HIF-1α/VEGF. Notably, NF-κB signaling is also regulated by lncRNAs at the transcriptional level in OC, as described in Section 2.2, illustrating that these molecules can control key oncogenic pathways through multiple regulatory layers.

### 2.5. Post-Translational Regulation of Protein Stability, Localization and Interactions

LncRNAs frequently regulate protein stability, localization, and activity through direct or indirect interactions [72,80]. Some lncRNAs bind target proteins and prevent their ubiquitination, thereby protecting them from proteasomal degradation. Others modulate post-translational modifications such as phosphorylation or SUMOylation, influencing protein function and interaction networks [73,81]. These mechanisms have been described in diverse cancer types, including OC, highlighting lncRNAs as key regulators of proteins’ fate.

Except for controlling protein stability, lncRNAs also regulate protein localization and interactions by acting as molecular scaffolds or guides (Figure 1). Specifically, these lncRNAs retain proteins within specific cellular compartments or promote their translocation to distinct subcellular locations in response to cellular signals [82,83]. In this manner, lncRNAs indirectly modulate protein activity, as localization to specific cellular compartments often represents a necessary step for subsequent post-translational modifications.

#### LncRNA-Mediated Regulation of Proteins in OC

In OC, multiple lncRNAs have been shown to regulate tumor progression by modulating protein stability and localization. In addition to its splicing-related role discussed above, MALAT1 is highly expressed in non-adherent OC spheres and cisplatin-resistant cells, where it promotes cancer stemness and chemoresistance. This lncRNA stabilizes the Yes-associated protein (YAP) and inhibits its nuclear translocation, which is necessary for its activity. By maintaining YAP stability and enhancing its function, MALAT1 contributes to the promotion of cancer stemness and resistance to chemotherapy. Restoring YAP expression can reverse the effects of MALAT1 knockdown, emphasizing the functional relevance of the MALAT1/YAP axis in OC biology.

Notably, Hippo-YAP signaling emerges as a recurrent post-translational target of lncRNAs in OC. In addition to MALAT1, UCA1 also regulates this pathway by enhancing the interaction between angiomotin (AMOT) and YAP, promoting YAP dephosphorylation and nuclear translocation (Figure 1I). This process leads to activation of YAP-dependent transcriptional programs that drive proliferation and tumor progression, establishing the UCA1/AMOT/YAP axis as another critical regulator of OC malignancy [84]. Together, these findings highlight YAP signaling as a key nodal point through which distinct lncRNAs promote aggressive OC phenotypes.

Beyond Hippo-YAP signaling, lncRNAs also modulate other oncogenic pathways through post-translational mechanisms. LINC00673 has been shown to promote cell proliferation, migration, and invasion while inhibiting apoptosis through interaction with the opioid growth factor receptor (OGFR). In vivo studies have shown that overexpression of LINC00673 leads to increased tumor growth in xenograft models, reinforcing its role in OC progression [85].

In contrast, the tumor-suppressive lncRNA GAS5 inhibits OC malignancy by reducing cell proliferation, migration, and promoting apoptosis. GAS5 exerts its effects by negatively regulating the stability of hnRNPK, a protein involved in RNA processing and cell survival. The binding of GAS5 to hnRNPK destabilizes the protein and suppresses the PI3K/AKT/mTOR signaling pathway, which is crucial for OC progression. Of note, this pathway is also regulated by other lncRNAs in OC through transcriptional and post-transcriptional mechanisms described in previous sections highlighting PI3K/AKT/mTOR signaling as a common target of lncRNA regulation across multiple levels. Through this axis, GAS5 limits the proliferative and metastatic capacity of OC cells, positioning it as a key modulator of OC biology [86].

Another lncRNA, CYMP-AS1, promotes OC progression by interacting with hnRNPM and inducing its translocation into the nucleus (Figure 1J). This step leads to the activation of the Wnt/β-catenin signaling pathway and drives malignant behaviors, such as enhanced cell proliferation, migration, invasion, and EMT. Knockout of CYMP-AS1 reduces these processes, highlighting its role in metastasis and progression of OC [87].

LncRNA-mediated post-translational regulation also contributes to therapy resistance and cellular stress adaptation. PART1, which is highly expressed in OC, plays a key role in regulating cellular processes related to drug resistance. Its inhibition induces cellular senescence by interacting with PHB2, a key mitophagy receptor. This interaction leads to the degradation of PHB2 and impairs mitophagy, disrupting the cellular maintenance mechanisms essential for optimal function. The depletion of PHB2 resulting from PART1 knockdown highlights its critical role in regulating mitophagy and cellular homeostasis, further emphasizing the importance of PART1 in OC progression [88].

FAM83-AS1 also contributes to OC progression and radioresistance by stabilizing the RNA-binding protein HuR. This stabilization enhances HuR activity, promoting OC cell migration, invasion, and resistance to radiation. Overexpression of HuR can reverse the effects of FAM83-AS1 silencing on metastasis and radioresistance, suggesting that FAM83-AS1 plays a key role in modulating the response to radiation and chemotherapy in OC [89]. Following, lncRNA LOC730101 played an essential role in attenuating drug resistance in OC. Mechanistically, LOC730101 specifically binds to BECN1 and inhibits the formation of autophagosome BECN1/VPS34 by reducing phosphorylation of BECN1, thereby inhibiting autophagy and promoting drug sensitivity in OC cells following treatment with cisplatin and PARP inhibitors [90]. 

Last but not least, H19, a well-known lncRNA for its implication in many cancers, plays a crucial role in cisplatin resistance in OC cells. H19 enhances cisplatin resistance by stabilizing erythroid 2-related factor 2 (Nrf2), a transcription factor that induces the transcription of Glutathione S-transferase Pi 1 (*GSTP1*). Activation of this pathway promotes glutathione synthesis, leading to cisplatin detoxification and reduced drug efficacy [91].

Summarizing, post-translational regulation is a major mechanism through which lncRNAs modulate OC biology. Multiple lncRNAs regulate YAP and PI3K/AKT signaling, highlighting these pathways as key post-translational targets and underscoring the critical role of lncRNAs in disease’s development and/or progression.

### 2.6. Additional Layers of Regulation: LncRNAs as Competing Endogenous RNAs

A widely studied function of cytoplasmic lncRNAs is their role as competing endogenous RNAs (ceRNAs) [92]. For this function, lncRNAs that contain miRNA response elements (MRE) act as molecular sponges, sequester miRNAs and reduce their availability to bind target mRNAs (Figure 1) [93,94,95,96]. Since miRNAs primarily suppress gene expression by inhibiting translation and promoting mRNA decay, lncRNA-mediated miRNA sponging generally results in the preservation of target mRNAs and, thus, increased protein production.

By modulating miRNA activity, lncRNAs indirectly regulate the expression of numerous protein-coding genes, thereby influencing critical biological processes [49,97,98]. Described in various cancer types, including OC, the ceRNA network introduces an additional layer of post-transcriptional regulation, highlighting the complexity and the extensive diversity of interactions that macromolecules can establish within the cell.

#### LncRNAs as ceRNAs in OC

Several lncRNAs have been reported to function as ceRNAs in OC, influencing disease development and progression. UCA1 is upregulated in response to lysophosphatidic acid (LPA) stimulation, and multiple experimental approaches support its interaction with the let-7 family of miRNAs. Specifically, RNA pull-down, cross-linking immunoprecipitation (CLIP), and luciferase reporter assays demonstrate that UCA1 can bind let-7 miRNAs, suggesting a ceRNA-based regulatory relationship that can inhibit their tumor-suppressive effects and promote tumor progression [99]. UCA1 has also been implicated in cisplatin resistance through regulation of the miR-143/FOSL2 axis. RNA immunoprecipitation (RIP) and luciferase reporter assays indicate that UCA1 and miR-143 interact, and that miR-143 can directly target the FOSL2 3′UTR [100]. Additionally, functional assays show that modulation of miR-143 levels affects cell proliferation in cisplatin-resistant and sensitive OC cells, supporting the functional relevance of the UCA1/miR-143 pathway in chemoresistance.

Another lncRNA involved in OC progression is LINC01123. It has been reported to interact with miR-516b-5p, suggesting a potential role in the regulation of angiogenesis. Dong et al. proposed that the binding of LINC01123 to miR-516b-5p upregulates vascular endothelial growth factor (VEGFA), which is essential for the formation of new blood vessels, thus supporting tumor growth and metastasis [101]. NEAT1 is another lncRNA involved in OC progression, through its interaction with miR-365. It is proposed that by binding to miR-365, NEAT1 upregulates fibroblast growth factor 9 (FGF9), enhancing angiogenesis and promoting tumor growth. This example illustrates how lncRNAs can regulate key factors involved in tumor vascularization, a critical aspect of cancer progression [102].

The tumor-suppressive lncRNA MEG3 has been implicated in OC through a proposed interaction with miR-205-5p (Figure 1G). Tao et al. suggest that MEG3 sequesters miR-205-5p, promoting the expression of PTEN and SMAD4, two key tumor-suppressor proteins that inhibit cell proliferation and survival [21]. LINC-PINT, which is downregulated in OC, has been reported to interact with miR-374a-5, suggesting a potential ceRNA-related regulatory relationship. Through this interaction, LINC-PINT is proposed to indirectly upregulates FOXO1, a transcription factor that suppresses EMT by promoting the expression of E-cadherin, an epithelial marker, and inhibiting vimentin, a mesenchymal marker [103].

LncRNA H19 has been reported to promote cisplatin resistance in OC through the H19/miR-29b-3p/STAT3 axis. Luciferase reporter, RNA pull-down, and Ago2-RIP assays support a direct interaction between H19 and miR-29b-3p, while functional experiments indicate that this interaction results in the upregulation of drug-resistance-associated proteins such as MRP1, P-glycoprotein (P-gp), LRP, and STAT3, which contribute to the inactivation of chemotherapeutic agents like carboplatin [104]. Lastly, the lncRNA SNHG1 is overexpressed in OC cell lines and has been associated with EMT and metastatic behavior, potentially through regulation of the miR-454/ZEB1 axis. Wu et al. suggest that SNHG1 binds to miR-454, resulting in the upregulation of ZEB1—a transcription factor that drives mesenchymal characteristics—and facilitating OC metastasis [98].

The ceRNA network represents a major post-transcriptional regulatory layer in OC, where multiple lncRNAs regulate key oncogenic pathways through miRNA sequestration. Although some ceRNA axes are well supported by OC-specific experiments, others remain preliminary and require further validation.

### 2.7. Structural Roles: Nuclear Architecture and Organization

Some lncRNAs function as architectural RNAs (arcRNAs) that serve as structural scaffolds for the assembly and maintenance of membraneless nuclear bodies, like paraspeckles, nuclear speckles, and other subnuclear compartments (Figure 1D) [105]. These RNAs are localized to specific nuclear sites and recruit proteins and other RNAs into RNP complexes, promoting the formation of dynamic nuclear condensates that concentrate regulatory factors involved in RNA processing and gene expression process [105,106].

Through interactions with RBPs, architectural lncRNAs contribute to nuclear architecture and genome organization. They can seed distinct nuclear bodies at specific genomic locations and contribute to the spatial compartmentalization of chromatin and regulatory machinery, influencing gene expression patterns both locally and genome-wide [107]. Disruption of these structural lncRNAs often compromises the integrity of nuclear bodies and can lead to altered gene regulation and cellular stress responses, underscoring their key structural and regulatory roles in nuclear function [108,109].

#### Nuclear Organization-Related LncRNAs in OC

NEAT1 plays a critical role in the DNA damage response and cellular stress adaptation, and its dysregulation can compromise nuclear body integrity and alter gene expression programs that support tumor cell survival and malignant progression [105]. Although both NEAT1_1 and NEAT1_2 are localized to paraspeckles, the formation and structural integrity of these nuclear bodies are strictly dependent on the long isoform, NEAT1_2 [109,110].

Previous studies across multiple cancer types, including OC, have demonstrated that NEAT1_2 contributes to the maintenance of genomic integrity by modulating ATR signaling. Activation of this pathway is essential for ensuring replication completion and preventing replication fork breakage in cells undergoing replication stress [108].

In platinum-resistant OC cells, CSTF3, a component of the cleavage stimulation factor complex, is frequently overexpressed. Functional studies have shown that knockdown of CSTF3 shifts NEAT1 processing toward increased production of the NEAT1_2 isoform by reducing premature cleavage and polyadenylation, thereby enhancing paraspeckle formation. Importantly, this isoform switch is associated with increased sensitivity of OC cells to platinum-based chemotherapy, highlighting the functional relevance of NEAT1 isoform regulation in therapeutic response [111]. Together, these findings establish NEAT1_2 as a key regulator of paraspeckle integrity, genome maintenance, and chemotherapeutic sensitivity in OC.

### 2.8. LncRNAs as Precursors of Small Regulatory RNAs

In addition to their direct regulatory and structural roles, many lncRNAs serve as precursor transcripts for small regulatory RNAs, including miRNAs, small nucleolar RNAs (snoRNAs), and Piwi-interacting RNAs (piRNAs). Certain lncRNAs harbor embedded miRNA sequences that are processed through canonical miRNA biogenesis pathways involving DROSHA and DICER, thus generating mature miRNAs with well-characterized roles in regulation of gene expression (Figure 1H) [112,113]. This mechanism has been primarily described in diverse cellular systems and other cancer types; however, studies with direct evidence for small RNA generation from lncRNAs in OC have not been reported yet.

Importantly, recent evidence demonstrates that many lncRNAs functioning as small RNA host transcripts also exert biological activities independent of the small RNAs they encode, participating in a wide range of regulatory processes. Notably, some lncRNAs that give rise to piRNAs are expressed in somatic tissues in which canonical piRNA biogenesis pathway is inactive, indicating that these transcripts may have additional biological roles beyond serving as precursors for the generation of small regulatory RNAs [114]. These findings highlight the dual roles of certain lncRNAs as both functional regulatory molecules and substrates for small RNA production, with RNA processing acting as a key post-transcriptional regulatory checkpoint.

#### Host LncRNAs in OC

As mentioned, direct evidence for small RNA generation from lncRNAs in OC is currently lacking. However, several lncRNAs that are highly relevant to OC have been shown to act as small RNA host genes in other malignancies. For example, the lncRNA H19 serves as the host transcript for the generation of miR-675, whose aberrant expression has been linked to enhanced proliferation, invasion, EMT, and chemoresistance through repression of tumor-suppressive pathways across multiple cancer cell lines [115,116].

Similarly, MIR503HG hosts miR-503 and miR-424, and has been reported to implicate in cell proliferation, invasion, metastasis, apoptosis, angiogenesis, and other cancer-related biological processes in OC [60]. Another example is PVT1, which gives rise to multiple miRNAs, including miR-1204, miR-1205, and miR-1206, and is associated with chemotherapy resistance in OC [117,118]. In addition, members of the small nucleolar RNA host gene (SNHG) family generate snoRNAs that can be further processed into smaller regulatory fragments. These molecules influence ribosome assembly, translation, and cellular stress responses, thereby involving in cancer development and progression [119].

In summary, several lncRNA-mediated mechanisms are supported by direct experimental evidence in OC, including PRC2/EZH2-dependent chromatin repression, transcriptional regulation, splicing modulation, and control of mRNA stability. Other mechanisms like enhancer-RNA activity, direct interaction with the translational machinery, and lncRNA-derived small RNA biogenesis, have been described in other cancer types but remain largely inferred in OC. These pathways therefore require further experimental validation in OC models.

## 3. Clinical Potential of LncRNAs in OC

LncRNAs have emerged as potential biomarkers for the diagnosis, prognosis, and treatment response prediction in OC. Early detection of OC remains challenging due to the limited sensitivity and specificity of conventional serum markers, resulting in most patients being diagnosed at advanced stages [4,120,121,122]. In this context, circulating and tissue-specific lncRNAs represent a promising and highly discriminative alternative, with transcriptomic studies demonstrating their ability to distinguish OC from normal ovarian epithelium with high accuracy [123,124]. Beyond diagnosis, aberrant lncRNA expression has been consistently associated with overall survival, progression-free survival, metastatic potential, and recurrence risk, with several lncRNA-based risk signatures validated as independent prognostic factors when integrated with clinical parameters like FIGO stage and tumor grade [125]. Furthermore, lncRNAs play a critical role in modulating response to platinum- and taxane-based chemotherapy by regulating apoptosis, DNA repair, EMT, and drug resistance pathways [72,74,121,126]. Specific lncRNAs have been linked to chemoresistance, highlighting their potential utility in predicting therapeutic outcomes and guiding personalized treatment strategies [118,126]. In this section, we discuss the emerging but still preliminary evidence supporting the potential clinical relevance of lncRNAs as diagnostic, prognostic, and predictive biomarkers in OC and their potential utility in clinical settings.

### 3.1. Limitations of Current Biomarkers for the Detection and Management of OC

Despite advances in surgical management and therapeutic approaches [127,128], OC remains associated with high mortality, mainly due to the lack of reliable biomarkers for early detection, prognostic stratification, and prediction of treatment response. Cancer Antigen 125 (CA125), the most widely used serum biomarker, exhibits substantial limitations, particularly for early-stage disease [129,130]. Only approximately 50% of patients with early-stage OC present with elevated CA125 levels, resulting in low sensitivity and limiting its utility as an effective screening tool [130]. Consequently, CA125 primarily distinguishes advanced-stage disease from healthy individuals and often fails to detect malignancy at a potentially curable stage, thereby leading to delayed diagnosis and unfavorable clinical outcomes [129]. The specificity of CA125 is also suboptimal in younger women (<50 years), in whom physiological variations related to the menstrual cycle, as well as benign gynecologic conditions (e.g., endometriosis) frequently lead to false-positive results [131,132,133]. Furthermore, increased CA125 levels are observed in a wide range of non-gynecologic malignancies, such as breast, gastrointestinal, pancreatic, and hepatobiliary cancers, further diminishing its positive diagnostic value [127,129,133,134].

The FDA-approved human epididymis protein 4 (HE4) is an additional serum biomarker for monitoring disease progression and recurrence in OC. Comparative analyses indicate that CA125 and HE4 have similar sensitivity, while HE4 shows higher specificity, particularly in differentiating benign from malignant pelvic masses. Since their sensitivity for early detection remains limited, both CA125 and HE4 are primarily used in combination rather than as standalone screening tools [135]. These limitations underscore the urgent need for more sensitive, specific, and biologically informative molecules, including lncRNAs, capable of enabling early diagnosis, improving risk stratification, and guiding personalized therapeutic strategies in OC.

### 3.2. LncRNAs as Diagnostic Biomarkers for OC Detection

Early diagnosis of OC can lead to improved survival; however, reliable biomarkers capable of detecting early-stage disease remain limited. Advances in transcriptomic profiling and molecular diagnostics have facilitated the identification of lncRNAs that are differentially expressed in OC tissues and body fluids, highlighting their potential utility as diagnostic biomarkers. High-throughput comparative gene expression analyses have revealed distinct lncRNA expression signatures that distinguish OC from normal ovarian epithelium with high sensitivity and specificity, supporting the promise of lncRNA-based approaches for more accurate OC detection.

Several lncRNAs exhibit associations with disease presence and severity, thereby suggesting possible diagnostic relevance. Multiple studies have demonstrated that lncRNAs such as LSINCT5, and BC041954 are significantly increased in OC tissues, whereas others, like BC200 and MEG3, are markedly downregulated, providing reliable molecular signatures for disease detection (Table 2) [136,137,138]. Consistent with these observations, lncRNA XIST was found to be overexpressed in OC tissues in a cohort of 98 patients, a finding that supports its diagnostic potential [139]. Similarly, elevated expression of MALAT1 has been consistently reported in OC tissues relative to normal ovarian epithelium across multiple independent studies [140,141].

In addition to tissue-based biomarkers, emerging evidence also supports the promising diagnostic value of exosome-derived lncRNAs. Circulating lncRNAs have gained increasing attention due to their remarkable stability in body fluids, which is attributed to their protection from RNase degradation through association with extracellular vesicles and exosomes [142]. Circulating lncRNAs therefore offer a minimally invasive approach for OC detection. Notably, exosomal MALAT1, secreted by OC cells, is significantly elevated in the serum of OC patients [143], while increased levels of exosomal aHIF have been reported in OC compared with healthy controls [144]. In line with this, serum levels of LOXL1-AS1 are significantly increased in patients with OC and have been associated with poor clinical outcomes, particularly in advanced-stage disease [145]. These findings suggest that exosome-associated lncRNAs can serve as highly specific and biologically informative biomarkers for OC detection.

Epigenetically regulated lncRNAs also represent a distinct and promising class of diagnostic biomarkers in OC. Methylation profiling of ten lncRNAs, including GAS5, HAND2-AS1, KCNK15-AS1, MAGI2-AS3, MEG3, SEMA3B-AS1, SNHG6, SSTR5-AS1, ZEB1-AS1, and ZNF667-AS1, demonstrated significantly increased promoter methylation in OC tissues compared with normal ovarian samples, accompanied by concordant downregulation of their expression. Specific methylation patterns were associated with metastatic dissemination, particularly to the peritoneum and omentum, and correlated with advanced clinical stage and tumor burden. Notably, partial reversal of hypermethylation in peritoneal metastases suggests dynamic epigenetic remodeling during metastatic progression. Collectively, these findings support lncRNA methylation signatures—especially SEMA3B-AS1, SSTR5-AS1, and ZNF667-AS1—as informative markers for disease stratification and outcome prediction in OC [136].

Although existing studies provide promising preliminary insights into the potential of lncRNAs as diagnostic biomarkers for OC, most evidence is derived from early-stage, and small-cohort analyses. Therefore, studies involving larger, independent cohorts are required to validate these findings and to determine the clinical utility of lncRNA-based methods for early OC detection.

### 3.3. LncRNAs as Prognostic Indicators

Among emerging molecular biomarkers, lncRNAs have gained considerable attention for their prognostic relevance in OC. Dysregulated lncRNA expression has been repeatedly associated with key determinants of disease outcome, including tumor aggressiveness, metastatic potential, chemoresistance, and reduced patient survival [146]. These associations underscore the potential utility of lncRNAs as indicators of disease progression and clinical prognosis in OC [147].

One of the most consistently reported oncogenic lncRNAs in OC is HOTAIR, whose expression is significantly elevated in OC tissues compared with normal ovarian epithelium. Multiple meta-analyses have demonstrated that high HOTAIR expression correlates with advanced FIGO stage and significantly shorter overall survival [148]. Similarly, PVT1 is frequently overexpressed in aggressive OC and has been linked to enhanced metastatic capacity, tumor recurrence, and resistance to chemotherapy. Elevated PVT1 expression is associated with unfavorable survival outcomes, underscoring its clinical relevance as a potential prognostic biomarker in OC [118]. MALAT1 is another lncRNA commonly upregulated in this malignancy and is associated with poor prognosis. Increased MALAT1 expression has been observed across multiple patient cohorts and is linked to enhanced migratory and invasive behavior, chemoresistance, and reduced survival [143,149,150].

In addition to these well-characterized lncRNAs, several others have been implicated in OC prognosis. Increased expression of LOC101927151 has been associated with poor prognosis, whereas reduced expression of LINC00861 and LEMD1-AS1 correlates with unfavorable outcomes [151]. Higher levels of PTPRD-AS1 and RP11-80H5.7 are similarly linked to reduced survival [152], while lower expression of MIR762HG and RP11-83A24.2 is associated with worse patient outcomes [18]. The tumor-suppressive lncRNA GAS5 is frequently downregulated in OC tissues, and its reduced expression correlates with worse disease-free and overall survival [153]. Likewise, elevated expression of BC041954 has been identified as an independent predictor of poor overall survival in OC patients [19]. Overexpression of LINC00511 and LINC01132 has also been associated with adverse clinical outcomes [18]. Furthermore, a multi-lncRNA panel comprising RUNX1-IT1, MALAT1, H19, HOTAIRM1, LOC100190986, and AL132709.8 has been linked to OC recurrence, emphasizing the prognostic value of combinatorial lncRNA expression patterns [154].

Additional lncRNAs continue to expand the prognostic landscape of OC. NEAT1 is frequently upregulated in OC and has been associated with poor prognosis; notably, it has been proposed as an independent predictor of overall survival in OC patients [155]. Similarly, UCA1 overexpression is associated with shorter overall survival and progression-free survival [22,156]. Other lncRNAs, including ANRIL, CCAT2, SPRY4-IT1, and ZFAS1, have been reported to correlate with aggressive tumor features and poor clinical outcomes, reflecting the growing number of lncRNAs implicated in OC prognosis [157,158,159].

Beyond individual lncRNAs, emerging multi-lncRNA prognostic signatures identified through high-throughput transcriptomic profiling and risk modeling may offer improved risk stratification than single biomarkers alone. By integrating the combined effects of multiple lncRNAs involved in EMT, apoptosis, and survival signaling pathways, these composite panels have demonstrated promising performance in stratifying patients into high- and low-risk groups with distinct survival outcomes. For example, Peng et al. proposed a prognostic model of 11 ferroptosis-related lncRNAs, including AC007848.1, AC010336.5, AL157871.2, AP001033.1, AC009403.1, AC068792.1, AC011445.1, AC093895.1, LINC01857, LINC00239 and AL513550.1, that accurately stratified OC patients according to survival risk [125]. These approaches highlight the complex and interconnected regulatory roles of lncRNAs in OC biology and their potential value in guiding prognosis and personalized clinical management.

### 3.4. LncRNAs Associated with Treatment Response and Drug Resistance

Chemoresistance remains the leading cause of treatment failure in OC. Accumulating evidence supports that the efficacy of therapeutic drugs is reduced due to the ability of lncRNAs to modify the expression of genes related to drug uptake, efflux, and metabolism [160]. Recent studies have identified numerous lncRNAs that are differentially expressed between chemoresistant and chemosensitive OC cells and patient samples, and have evaluated their functional roles in resistance to commonly used OC therapies (Figure 2) [161,162].

Several lncRNAs have been implicated in the regulation of drug efflux and survival pathways associated with chemoresistance. For example, elevated expression of LINC01118 and WDFY3-AS2 has been observed in OC cell lines and are associated with resistance to paclitaxel (Tax) and cisplatin, respectively. These lncRNAs exert their effects, at least in part, by acting as ceRNAs [163,164]. Similarly, increased levels of the lncRNAs PRLB and SNHG22 have been linked to diminished responsiveness to Tax- and cisplatin-based chemotherapy, with higher expression correlating with unfavorable treatment outcomes [165,166]. These findings highlight a subset of lncRNAs whose overexpression may serve as indicators of intrinsic or acquired chemoresistance, underscoring their exploit for clinical utility.

Alterations in drug metabolism have also been associated with lncRNA dysregulation in OC. Reduced expression of PVT1 has been linked to increased sensitivity to doxorubicin in OC cell models, while multiple studies have associated the elevated H19 and UCA1 expression with cisplatin and/or Tax resistance [91,100,167,168,169]. In addition, NEAT1 and CCAT1 are frequently upregulated in chemoresistant OC phenotypes and have been correlated with reduced sensitivity to platinum- and Tax-based therapies [170,171]. MALAT1, a lncRNA widely implicated in OC progression, is also upregulated in cisplatin-resistant OC and its knockdown enhances chemosensitivity of OC cells to cisplatin, and its knockdown enhances chemosensitivity to cisplatin, further supporting its role in chemotherapy resistance [172].

Among lncRNAs associated with chemoresistance, HOTAIR is one of the most extensively studied. Its overexpression has been linked to cisplatin- and Tax-based resistance, effects that accomplishes through its function as a ceRNA [173,174]. Experimental and clinical evidence suggests that high HOTAIR expression promotes cancer cell survival during chemotherapy, supporting its potential value as a predictive biomarker of treatment response.

In contrast to chemoresistance-promoting lncRNAs, several tumor-suppressive lncRNAs have been associated with enhanced treatment sensitivity in OC. LINC00312, LINC01508, and LINC01125 are downregulated in chemoresistant OC cells and patient tissues, and their reduced expression correlates with diminished responsiveness to cisplatin-based chemotherapy (Table 2) [175,176]. Similarly, GAS5 has been reported to be expressed at lower levels in cisplatin-resistant OC tissues compared with sensitive tumors, and restoration of its expression has been associated with improved treatment response [59]. Other lncRNAs, including FER1L4 and SNHG5, have also been linked to increased chemosensitivity and apoptotic responsiveness when expressed at higher levels [177,178].

Although resistance to chemotherapy has been the primary focus of lncRNA research in OC, emerging evidence also involves lncRNAs in radiotherapy response. Notably, elevated expression of FAM83H-AS1 has been associated with radioresistance in multiple OC cell lines and tissue samples, whereas its downregulation enhances radiosensitivity [89]. These findings suggest that lncRNAs may also influence DNA damage responses and survival pathways relevant to radiation therapy, although this area remains underexplored in OC [179].

## 4. Advancing lncRNA Research in OC: Emerging Technologies and Future Directions

The future of lncRNA research in OC is tightly linked to continued advances in sequencing technologies, particularly the transition from next-generation sequencing (NGS) to third-generation sequencing (TGS). While NGS platforms revolutionized genomics and transcriptomics by enabling high-throughput sequencing at relatively low cost compared to Sanger sequencing, their reliance on short read lengths has limited the comprehensive characterization of structurally complex RNA molecules [23,180]. This limitation is especially pronounced for lncRNAs, which are long in length and frequently derive from complex genomic loci.

Short-read NGS platforms generally produce reads ranging from 50 to 600 base pairs, requiring *in silico* assembly to reconstruct full-length transcripts [180,181]. For lncRNAs, this process is particularly error-prone due to alternative splicing, overlapping transcriptional units, and heterogeneous isoform structures. Moreover, PCR amplification—an essential step in NGS workflows—introduces sequence bias, reduces quantitative accuracy, and prevents direct detection of native epigenetic and epitranscriptomic modifications.

As cancer research increasingly demands a more complete understanding of transcript isoform diversity, RNA modifications, and transcriptome complexity, it has become clear that short-read sequencing alone is insufficient [182,183]. This need has driven the development and adoption of TGS technologies, which enable single-molecule, real-time sequencing and the generation of long reads [23]. The two most widely used TGS platforms—Pacific Biosciences’ Single-Molecule Real-Time (SMRT) sequencing and Oxford Nanopore Technologies’ nanopore sequencing [182]—have significantly reshaped transcriptomic analysis by generating sequencing reads with average lengths of approximately 10 kb, often spanning full-length transcripts [23,184]. As a result, TGS platforms are becoming the most suitable option for whole-transcriptome sequencing, enabling more efficient and accurate exploration of complex transcriptomes without reliance on specialized or error-prone transcript assembly algorithms [23,180,185].

**Figure 3:**
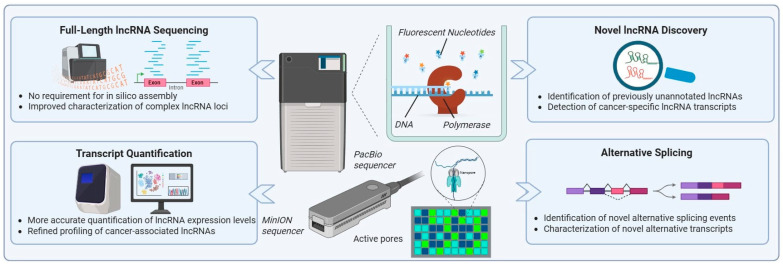
Long-read sequencing technologies for lncRNA research. The figure represents the long-read sequencing platforms introduced by PacBio and Oxford Nanopore Technologies (MinION), and their applications in lncRNA research. These technologies enable full-length lncRNA sequencing without the need for *in silico* assembly, allowing accurate characterization of complex lncRNA loci. Key applications include precise transcript quantification, identification of previously unannotated lncRNAs, detection of cancer-specific lncRNA isoforms, and comprehensive analysis of alternative splicing events. The use of long-read sequencing can significantly improve the study of lncRNA transcriptome in OC, facilitating the discovery of novel OC-related signatures.

For lncRNA research, the implications of these capabilities are substantial. TGS platforms can directly sequence full-length transcripts, enabling precise delineation of exon–intron structures and alternative splicing events without reliance on *in silico* assembly (Figure 3) [186,187]. This is particularly critical for lncRNAs, whose functional roles are often isoform-specific and tightly linked to transcript structure. The importance of these technologies has been demonstrated in research works across multiple cancer types, where long-read sequencing approaches have substantially refined lncRNA annotations (Figure 3). Notably, studies in breast and hepatocellular carcinoma have shown that long-read RNA sequencing can reveal widespread transcript structural variations among cancer-associated lncRNAs, highlighting the prevalence of isoform-level regulation in tumor biology [186,188]. In these studies, TGS enabled the direct resolution of complex splice variants and transcript boundaries, leading to the identification of lncRNA isoforms with distinct regulatory potential that had been misassembled or entirely missed by short-read approaches. These findings underscore the extent to which conventional NGS approaches may underestimate lncRNA diversity in cancer.

Beyond transcript structure, TGS platforms offer unique advantages for investigating RNA modifications through direct RNA sequencing (Figure 3) [189,190,191]. Long-read nanopore-based studies in cancer models have demonstrated the feasibility of detecting epitranscriptomic marks, such as N6-methyladenosine, on lncRNA transcripts. These studies provide evidence that RNA modification patterns differ between tumor and normal tissues and may contribute to cancer-specific regulation of lncRNA stability and function [192,193,194]. Importantly, such high-throughput information remains largely inaccessible to short-read NGS methodologies.

In OC, where transcriptomic heterogeneity and therapeutic resistance remain major clinical challenges, TGS-based approaches can be exploited to uncover previously unrecognized layers of lncRNA complexity. To date, long-read sequencing has revealed unannotated transcripts, cancer-specific splicing patterns, gene fusions, and novel SVs—including large insertions, deletions, and complex rearrangements—in OC that are not detectable using short-read data [195,196,197,198]. These findings have important implications for biomarker discovery, as full-length transcript resolution enables more accurate evaluation of isoform-specific expression patterns associated with disease subtype, prognosis, and treatment response [186]. Furthermore, improved characterization of lncRNA regulatory networks may facilitate the identification of new therapeutic targets and enhance understanding of mechanisms underlying tumor progression and drug resistance.

Looking ahead, broader adoption of TGS in OC research is expected to substantially advance the field by enabling accurate characterization of full-length lncRNA transcripts, isoform-specific expression patterns, and RNA modification landscapes. Hybrid approaches that combine the accuracy of NGS with the structural resolution of TGS may further enhance lncRNA annotation and functional analyses [195,198]. Overall, TGS technologies promise to transform lncRNA research and accelerate the translation of lncRNA discoveries into clinical applications.

## 5. CRISPR-Cas13 System for Silencing Oncogenic lncRNAs in OC: Emerging Therapeutic Potential

The CRISPR-Cas13 system, a cutting-edge RNA-targeting approach, has emerged as a powerful tool for modulating gene expression, advancing functional studies in cancer research. Unlike the widely used CRISPR/Cas9 system, which edits DNA [199], CRISPR-Cas13 cleaves single-stranded RNA, enabling reversible and non-permanent modulation of gene activity [200]. This unique feature makes Cas13 particularly attractive for applications where preserving DNA integrity is critical. Despite its theoretical suitability for targeting oncogenic lncRNAs, the CRISPR-Cas13 system has not yet been evaluated for lncRNA-directed therapeutic applications in OC, and the evidence supporting its use for RNA silencing remains indirect. However, owing to its capacity for precise RNA degradation, CRISPR-Cas13 represents a promising tool for controlling tumor growth, metastasis, and chemoresistance [28], highlighting its potential for future integration into OC therapeutic approaches.

Cas13 comprises multiple RNA-guided endonuclease subtypes, including Cas13a, Cas13b, Cas13d, and Cas13x, which function in complex with custom-designed guide RNAs (gRNAs) [200,201]. Mechanistically, the gRNA directs Cas13 to complementary RNA targets, and upon binding, Cas13 cleaves the transcript, effectively reducing its cellular levels and silencing its function [201,202]. Since Cas13 does not interact with DNA, it minimizes the risk of off-target genetic mutations and offers a safer and highly adaptable approach for therapeutic intervention in OC [203].

Several oncogenic lncRNAs, including HOTAIR [97], H19 [91,104], and MALAT1, have been validated in numerous studies as key contributors to OC development and progression [126,204]. These lncRNAs drive critical oncogenic processes like cell proliferation, migration, invasion, EMT, and resistance to apoptosis, highlighting their crucial role in tumor biology [141,149,205]. The CRISPR-Cas13 system offers a unique advantage for targeting these transcripts at the RNA level. This allows selective silencing of specific lncRNA isoforms while preserving other alternative transcripts derived from the same genomic locus. In contrast, CRISPR-Cas9 system introduces permanent insertions or deletions in the DNA [199,206,207], disrupting the entire gene and abolishing the expression of all associated transcripts. This transcript-specific targeting makes Cas13 a more precise and versatile tool for potential therapeutic applications in OC.

Although the idea of targeting lncRNAs with CRISPR-Cas13 in cancer therapeutics is something that has not happened yet, the successful translation of Cas13-based RNA therapies in other diseases provides compelling proof-of-concept for this platform. Notably, HuidaGene Therapeutics initiated at the end of 2024 the first-in-human clinical programs employing CRISPR-Cas13 RNA-editing technology, underscoring the feasibility, safety, and therapeutic potential of targeted RNA degradation in humans. The HERO trial (NCT06615206) evaluates HG204, a Cas13-based RNA therapy designed to selectively degrade overexpressed MECP2 mRNA in patients with MECP2 Duplication Syndrome, a severe neurodevelopmental disorder, with early clinical data indicating favorable tolerability and preliminary therapeutic benefit [208]. In parallel, the SIGHT-I (NCT06031727) and BRIGHT (NCT06623279) trials are assessing HG202, a Cas13-based therapeutic targeting VEGF-A mRNA for the treatment of neovascular age-related macular degeneration, marking the first Cas13 RNA-editing therapy cleared by the FDA for human testing [209].

Importantly, these advances are particularly relevant to OC therapy, as lncRNAs share several features with mRNAs, including transcription by RNA polymerase II, the presence of a 5′ cap, polyadenylation at the 3′ end, and often, multiple exons [210,211,212]. Consequently, strategies that efficiently target pathogenic mRNAs can, in principle, be adapted to selectively silence disease-associated lncRNAs in OC treatment.

Notably, CRISPR-Cas13 system represents a promising approach for modulating oncogenic lncRNAs in OC therapy. By enabling precise and selective degradation of pathogenic RNA transcripts without altering genomic DNA [203], Cas13 offers a safer and more controllable strategy for therapeutic intervention [28]. This RNA-specific targeting preserves genomic integrity while allowing reversible modulation of disease-associated lncRNAs, conferring a significant advantage over DNA-editing approaches.

## 6. Conclusions

Over the past decade, lncRNAs have emerged as critical regulators of OC biology, exerting control over gene expression across multiple regulatory layers [72]. As highlighted in this review, lncRNAs modulate chromatin architecture, transcription, RNA processing, mRNA stability, protein function, and intercellular signaling through diverse mechanisms. Their ability to regulate gene expression at epigenetic, transcriptional, post-transcriptional, and post-translational levels positions lncRNAs as key drivers of OC initiation and progression, metastasis, and therapeutic resistance [36].

Accumulating mechanistic evidence has established lncRNAs as important molecules with critical roles in oncogenic and tumor-suppressive pathways. Aberrant lncRNA expression contributes to key malignant phenotypes, including EMT, cancer stemness, sustained proliferation, angiogenesis, immune evasion, and resistance to platinum- and taxane-based therapies. These diverse effects underscore the remarkable complexity of lncRNA-mediated regulatory networks and their capacity to reprogram cellular states in response to oncogenic signals and treatment-induced selective pressure.

Beyond their biological significance, lncRNAs have emerged as potential biomarkers in OC. Tissue-specific, circulating, and epigenetically regulated lncRNAs have shown diagnostic and prognostic relevance based on preliminary data and early-stage studies. Moreover, multi-lncRNA signatures derived from high-throughput transcriptomic analyses provide robust risk stratification and prognostic accuracy, supporting their potential integration into precision oncology frameworks for OC [125].

Looking ahead, continued technological innovations are expectedto substantially advance the field of lncRNA research. In particular, long-read sequencing platforms offer unprecedented opportunities to resolve full-length lncRNA transcripts, characterize isoform diversity, and detect epitranscriptomic modifications with high accuracy [23]. These capabilities are essential for elucidating isoform-specific functions and identifying previously unannotated lncRNAs with potential diagnostic or therapeutic relevance. As the transcriptomic complexity of OC becomes increasingly apparent, long-read sequencing and integrative multi-omics strategies will be indispensable for translating lncRNAs into clinical applications.

In parallel, emerging RNA-targeting therapeutic strategies—most notably the CRISPR-Cas13 system—offer a promising approach for directly targeting lncRNAs. Unlike DNA-editing methods, Cas13 can achieve precise, reversible, and transcript-specific silencing of oncogenic lncRNAs without permanent genomic alterations [28,202]. The growing success of Cas13-based therapies for non-cancerous indications provides compelling proof-of-concept for their future application in OC. By enabling precise, reversible, and transcript-specific modulation, RNA-targeting therapies are about to overcome critical limitations of current treatment modalities and offer new avenues in OC therapeutics.

In conclusion, lncRNAs represent a constitutive regulatory layer in OC biology with profound implications for diagnosis, prognosis, and therapy [129]. Continued integration of mechanistic studies, advanced sequencing technologies, and innovative RNA-targeting approaches will be essential to fully exploit the potential of lncRNAs in clinical settings, facilitating early detection of OC and improving patient outcomes.

## Figures and Tables

**Table 1:** LncRNAs in OC. The table summarizes the discussed lncRNAs’ functional role, mechanism of action, key biological effects, experimental evidence level, and potential clinical relevance in OC.

LncRNA	Functional Role	Mechanism of Action	Key Biological Effects	Evidence	Potential Clinical Relevance
HOTAIR	Oncogenic	PRC2/EZH2 and LSD1 recruitment	Proliferation, invasion, chemoresistance	In vitro, in vivo	Prognostic; predictive
LINC00511	Oncogenic	EZH2-mediated p21 repression	Cell cycle progression	In vitro	Prognostic
TP73-AS1	Oncogenic	EZH2 recruitment to p21 promoter	Proliferation, invasion	In vitro	Prognostic
LINC01210	Oncogenic	EZH2-mediated KLF4 repression	Migration, invasion	In vitro	-
LINC00702	Oncogenic	EZH2-mediated KLF2 repression	Proliferation	In vitro	-
GAS5	Tumor suppressor	Transcriptional activation; hnRNPK destabilization	Apoptosis, chemosensitivity	In vitro	Prognostic; predictive
MIR503HG	Tumor suppressor	SPI1-mediated transcriptional repression	EMT inhibition	In vitro	Diagnostic; prognostic
FAM225B	Tumor suppressor	DDX17-mediated PDIA4 transcription	Growth suppression	In vitro	Diagnostic
LINC01320	Oncogenic	PURB-mediated DDB2 repression	Metastasis	In vitro	-
TMPO-AS1	Oncogenic	E2F6 displacement; *LCN2* activation	Tumor growth, angiogenesis	In vitro	-
RFPL1S-202	Tumor suppressor	DDX3X-mediated m6A/STAT1 inhibition	Chemosensitivity, metastasis suppression	In vitro	-
LINC00176	Oncogenic	Molecular scaffold facilitating BCL3–NF-κB p50 interaction	NF-κB activation	In vitro	-
MALAT1	Oncogenic	Alternative splicing; YAP stabilization	Stemness, anoikis resistance	In vitro, in vivo	Diagnostic; prognostic; predictive
HCP5	Oncogenic	PTBP1 splicing regulation	Cell survival, cell cycle	In vitro	-
PANDAR	Oncogenic	SFRS2–p53 interaction	Cisplatin resistance	In vitro, in vivo	Predictive
LOXL1-AS1	Oncogenic	*BRIP1* mRNA stabilization	Proliferation, EMT	In vitro, in vivo	Diagnostic; prognostic
TLR8-AS1	Oncogenic	*TLR8* mRNA stabilization	NF-κB activation, resistance	In vitro, in vivo	Prognostic
HOTTIP	Oncogenic	HIF-1α stabilization	Hypoxia adaptation, angiogenesis	In vitro, in vivo	Prognostic
UCA1	Oncogenic	AMOT-YAP interaction; ceRNA	EMT, proliferation, resistance	In vitro, in vivo	Prognostic; predictive
LINC00673	Oncogenic	OGFR interaction	Proliferation, invasion	In vitro, in vivo	-
CYMP-AS1	Oncogenic	hnRNPM nuclear translocation	Wnt/β-catenin activation	In vitro, in vivo	-
PART1	Oncogenic	PHB2 degradation; mitophagy inhibition	Drug resistance	In vitro, in vivo	Predictive; prognostic
FAM83-AS1	Oncogenic	HuR stabilization	Metastasis, radioresistance	In vitro, in vivo	Predictive
LOC730101	Tumor suppressor	BECN1/VPS34 autophagy inhibition	Drug sensitivity	In vitro, in vivo	Predictive
H19	Oncogenic	Nrf2 stabilization; ceRNA	Cisplatin resistance	In vitro, in vivo	Predictive; prognostic
MEG3	Tumor suppressor	ceRNA (PTEN/SMAD4)	Growth inhibition	In vitro	Diagnostic; prognostic
LINC-PINT	Tumor suppressor	ceRNA-mediated FOXO1 activation	EMT suppression	In vitro	Prognostic
LINC01123	Oncogenic	ceRNA (VEGFA upregulation)	Angiogenesis	In vitro	-
SNHG1	Oncogenic	ceRNA (miR-454/ZEB1)	EMT, metastasis	In vitro	Prognostic
NEAT1	Oncogenic	Paraspeckle formation; ATR signaling	DNA damage tolerance	In vitro, in vivo	Prognostic; predictive
PVT1	Oncogenic	miRNA host	Chemoresistance	In vitro	Prognostic; predictive

**Table 2:** LncRNAs with potential clinical relevance in OC. Summary of lncRNAs assessed exclusively for their diagnostic, prognostic, or predictive value in OC.

LncRNA	Potential Clinical Relevance
LSINCT5	Diagnostic
BC041954	Diagnostic; prognostic
BC200	Diagnostic
XIST	Diagnostic
aHIF	Diagnostic
LINC00861	Prognostic
LEMD1-AS1	Prognostic
LOC101927151	Prognostic
PTPRD-AS1	Prognostic
RP11-80H5.7	Prognostic
MIR762HG	Prognostic
RP11-83A24.2	Prognostic
RUNX1-IT1	Prognostic
HOTAIRM1	Prognostic
LOC100190986	Prognostic
AL132709.8	Prognostic
LINC01118	Predictive
WDFY3-AS2	Predictive
PRLB	Predictive
SNHG22	Predictive
CCAT1	Predictive
ANRIL	Prognostic
CCAT2	Prognostic
SPRY4-IT1	Prognostic
ZFAS1	Prognostic
FER1L4	Predictive
SNHG5	Predictive
LINC00312	Predictive
LINC01508	Predictive
LINC01125	Predictive
FAM83H-AS1	Predictive

## Data Availability

No new data were created or analyzed in this study. Data sharing is not applicable to this article.
